# 

**Published:** 1879-03-20

**Authors:** 


RECIPTS.—Being pressed for space, we have omitted receipts of subscriptions for the past month. They will appear in our next.

